# Fluoxetine Treatment Decreases Cardiac Vagal Input and Alters the Serotonergic Modulation of the Parasympathetic Outflow in Diabetic Rats

**DOI:** 10.3390/ijms23105736

**Published:** 2022-05-20

**Authors:** Mónica García-Domingo, José Ángel García-Pedraza, Juan Francisco Fernández-González, Cristina López, María Luisa Martín, Asunción Morán

**Affiliations:** 1 Laboratorio de Farmacología, Departamento de Fisiología y Farmacología, Facultad de Farmacia, Universidad de Salamanca, 37007 Salamanca, Spain; joseagp@usal.es (J.Á.G.-P.); juanfdezglez@usal.es (J.F.F.-G.); peztinalop@usal.es (C.L.); marisam@usal.es (M.L.M.); amoran@usal.es (A.M.); 2 Instituto de Investigación Biomédica de Salamanca (IBSAL), Paseo San Vicente 58-182, 37007 Salamanca, Spain

**Keywords:** 5-HT_1D_ receptor, 5-HT_7_ receptor, bradycardia, cardiac parasympathetic neurotransmission, depression, diabetes, fluoxetine, heart diseases

## Abstract

Comorbid diabetes and depression constitutes a major health problem, worsening associated cardiovascular diseases. Fluoxetine’s (antidepressant) role on cardiac diabetic complications remains unknown. We determined whether fluoxetine modifies cardiac vagal input and its serotonergic modulation in male Wistar diabetic rats. Diabetes was induced by alloxan and maintained for 28 days. Fluoxetine was administered the last 14 days (10 mg/kg/day; p.o). Bradycardia was obtained by vagal stimulation (3, 6 and 9 Hz) or i.v. acetylcholine administrations (1, 5 and 10 μg/kg). Fluoxetine treatment diminished vagally-induced bradycardia. Administration of 5-HT originated a dual action on the bradycardia, augmenting it at low doses and diminishing it at high doses, reproduced by 5-CT (5-HT_1/7_ agonist). 5-CT did not alter the bradycardia induced by exogenous acetylcholine. Decrease of the vagally-induced bradycardia evoked by high doses of 5-HT and 5-CT was reproduced by L-694,247 (5-HT_1D_ agonist) and blocked by prior administration of LY310762 (5-HT_1D_ antagonist). Enhancement of the electrical-induced bradycardia by 5-CT (10 μg/kg) was abolished by pretreatment with SB269970 (5-HT_7_ receptor antagonist). Thus, oral fluoxetine treatment originates a decrease in cardiac cholinergic activity and changes 5-HT modulation of bradycardic responses in diabetes: prejunctional 5-HT_7_ receptors augment cholinergic-evoked bradycardic responses, whereas prejunctional 5-HT_1D_ receptors inhibit vagally-induced bradycardia.

## 1. Introduction

Comorbid diabetes mellitus and depression is often under-recognised and a challenging clinical problem, with growing numbers since the year 2020 due to the global pandemic [1,2,3]. The appearance of cardiovascular autonomic neuropathy (CAN) affects up to 90% of diabetes mellitus patients and increases mortality by two- to five-fold compared to diabetic patients without CAN [4,5]. It is a microvascular complication that results from damage to the nerves that innervate the heart and augments the risk of chronic kidney disease, cardiac arrhythmias, myocardial dysfunction, silent myocardial ischemia, major cardiovascular events, and sudden death [6]. Acetylcholine (ACh) release from vagus nerve is responsible of the parasympathetic regulation of heart rate (HR). Previous studies have shown that regulation of HR by the parasympathetic nervous system is altered in type 1 diabetes mellitus (T1DM) and that this occurs in association with impaired responsiveness to parasympathetic nervous system agonists in the sinoatrial node [7,8]. Moreover, the earliest manifestations of CAN tend to be associated with parasympathetic denervation, with consequent early augmentation of sympathetic tone [9]. These complications may be potentiated by depression, which also affects immune, neuroendocrine and neurotransmission systems. In fact, alteration in monoamines (serotonin and noradrenaline), among other factors, constitutes one of the abnormalities documented in diabetic patients and in animal models that could explain the association between depression and diabetes [10]. Selective serotonin reuptake inhibitors (SSRIs), such as fluoxetine, are a common treatment in depression [11]. Apart from their central neuronal effect, SSRIs affect the cardiovascular homeostasis, showing both positive and negative consequences [12,13,14] and playing a significant role in regulating autonomic nervous system [15] that can also lead to alterations in autonomic diabetic neuropathy.

5-hydroxytriptamine (5-HT) has been shown to play an important role in the regulation of the autonomic nervous system at the cardiac level. In pithed rats, 5-HT regulates sympathetic cardiac outflow [16,17] and also modulates vagal outflow to the heart via 5-HT_2_ and 5-HT_3_ receptor activation [18]. However, experimental T1DM, as well as pharmacological 5-HT modulation by the antagonism of 5-HT_2_ receptor, modifies the serotonergic effect on both sympathetic and parasympathetic cardiac discharge [19,20,21,22,23,24]. Furthermore, our group has demonstrated that, in naïve animals, the chronic treatment with a SSRI (fluoxetine) modifies the serotonergic modulation of vagal bradycardia, exerting an inhibitory action of the vagal cardiac input due to activation of prejunctional 5-HT_1D_ receptors [25].

Taking into account that: (1) currently, SSRI are usually the treatment for acute or chronic stress, anxiety or depression, which are pathologies that have become even more highly prevalent in diabetic patients [3]; (2) diabetic autonomic neuropathy contributes, in these patients, to an increased propensity for complex ventricular arrhythmias and high cardiac mortality rate [4,26]; and that (3) 5-HT modulates parasympathetic cardiac outflow in normoglycaemic, diabetic and fluoxetine-treated (FT) normoglycaemic rats, a further insight in the mechanisms involved in cardiac regulation during diabetes and the role of fluoxetine treatment on it may help to better understand the mechanisms underlying the interrelation between depression and CAN in diabetic patients. In this line, the goal of this study was to establish whether chronic treatment with fluoxetine may affect the vagal input to the heart and also determine the influence of serotonergic system on cardiac parasympathetic neurotransmission in diabetic rats, establishing the 5-HT receptors involved in peripheral serotonergic modulation of cardiac vagal drive in FT diabetic rats.

## 2. Results

### 2.1. Systemic Haemodynamic Parameters

The induction of a diabetic state using alloxan originated a significant augmentation of glycaemia at day 2 after induction (503 ± 8 mg/dL at day 2 versus 98 ± 3 mg/dL at day 0; *p* < 0.05 versus day 0, that was maintained until day 14 (491 ± 10 mg/dL; *p* < 0.05 versus day 0). In the diabetic control group (*n* = 5), this hyperglycaemia remained stable until day 28 (488 ± 12 mg/dL; *p* < 0.05 versus day 0). The chronic administration of fluoxetine (*n* = 115) significantly diminished glucose levels in diabetic animals (270 ± 7 mg/dL; *p* < 0.05 versus day 0, day 14 and diabetic control group at day 28), even though glycaemia did not return to basal values, as already described [27]. In relation to body weight gain, as previously shown by our group [27], the chronic administration of fluoxetine restricted body weight increase (281 ± 10 g at day 0; 339 ± 5 g at day 28 in diabetic control group—*p* < 0.05 versus day 0; and, 287 ± 4 g at day 28 in FT diabetic group—*p* < 0.05 versus day 28 of diabetic control group).

After anaesthesia and pithing, the basal mean blood pressure (MBP) and HR in control diabetic rats were 51 ± 3 mm Hg and 268 ± 7 beats/min, respectively, and did not vary after i.v. administration of saline. In FT diabetic rats, these parameters were 52 ± 5 mm Hg and 264 ± 8 beats/min, respectively. These values (in FT diabetic rats) were not significantly changed after i.v. bolus administration of saline or 5-HT receptor type/subtype agonists (5-CT, 8-OH-DPAT, CGS-12066B, L-694,247, 1-PBG) or 5-HT receptor antagonists (LY310762 and SB269970). Remarkably, i.v. bolus of increasing doses of 5-HT and α-methyl-5-HT originated an augmentation of MBP in a dose-dependent manner, that returned to basal values in less than 1 min (Table 1).

### 2.2. Alterations in the HR by Electrical Vagal Stimulation or Exogenous ACh

Stimulation of the vagus nerve instantly induced a frequency-dependent bradycardia that was restored once the electrical stimulation finished. The electrically-induced reduction in heart rhythm were: −28.6 ± 2.5, −54.2 ± 5.1 and −88.8 ± 7.3 and −7.1 ± 2.6, −16.0 ± 3.1 and −28.9 ± 1.7 beats/min (control stimulation-response (S-R) curve) at the 3, 6 and 9 Hz frequencies, in control diabetic group and FT diabetic rats, respectively. During the entire experiment, MBP was not substantially altered, as already shown [20,21,25]. These responses were reproducible, as they did not essentially change after i.v. injection of physiological solution (1 mL/kg) in diabetic control group (−29.3 ± 1.8, −57.2 ± 3.5 and −90.1 ± 6.8 beats/min at the 3, 6 and 9 Hz frequencies, respectively) and in FT diabetic rats (Table 2).

The administration of increasing doses of ACh (1, 5 and 10 μg/kg; i.v.) in FT diabetic rats originated dose-dependent bradycardic responses (−41.2 ± 1.5, −51.4 ± 3.7, −67.5 ± 7.9 beats/min; control dose-response (D-R) curve). These bradycardic effects remained stable after bolus i.v. administration of saline (1 mL/kg; Table 3).

### 2.3. Effect of 5-HT on Electrically-Induced Bradycardia in FT Diabetic Rats

In FT diabetic animals, intravenous administration of low doses of 5-HT (10 and 50 μg/kg) caused a significant increase in the vagally induced bradycardia at all stimulation frequencies tested, except for 5-HT 50 μg/kg (3 and 6 Hz) (Table 2), whereas high doses of 5-HT (100 and 200 μg/kg) resulted in a significant decrease of the vagally induced bradycardia (Table 2). 

### 2.4. Role of 5-HT Receptor Serotonergic Agonists on the Vagally Induced Bradycardia in FT Diabetic Rats

**Figure 1:**
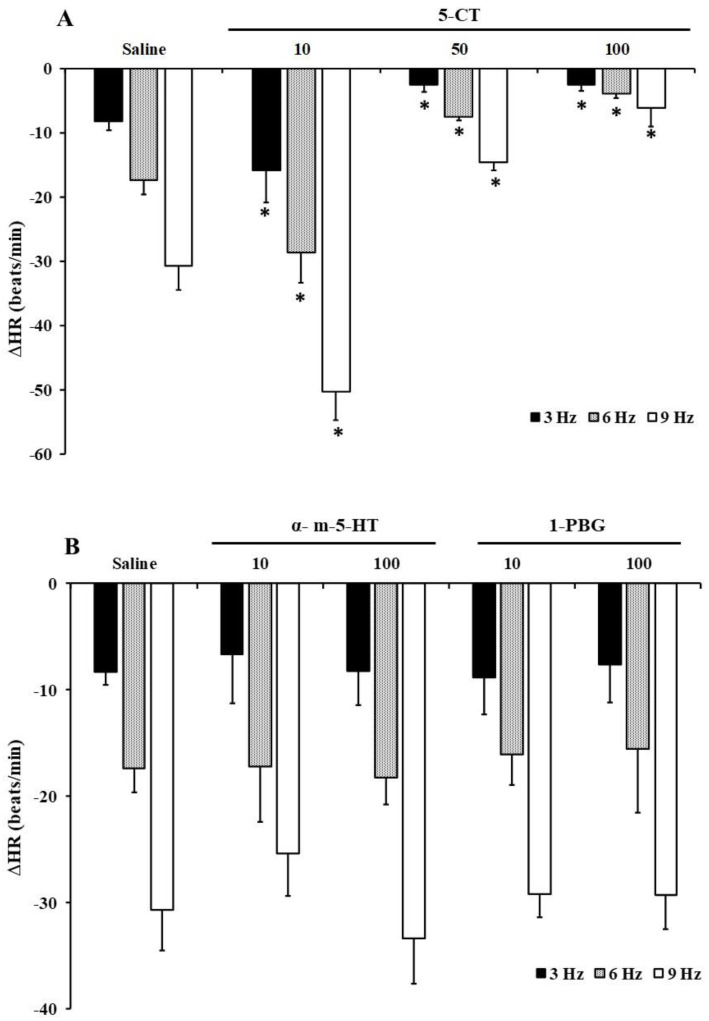
Effect of 5-HT receptor type agonists on the vagally-induced bradycardic responses in fluoxetine-treated diabetic rats. Effect of i.v. bolus injections of saline (1 mL/kg) and (**A**) 5-CT (10, 50 and 100 μg/kg; *n* = 5 for each dose) or (**B**) α-methyl-5-HT (α-m-5-HT) and 1-phenylbiguanide (1-PBG) (10 and 100 μg/kg each agonist; *n* = 5 for each treatment and dose) on the decreases in heart rate (ΔHR) evoked by electrical vagal stimulation (3, 6 and 9 Hz) in fluoxetine-treated diabetic pithed rats. * *p* < 0.05 versus saline.

In FT diabetic rats, the administration of the 5-HT_1/7_ agonist, 5-CT, has a dual effect, inducing a potentiation of the electrically-induced bradycardia at low doses (10 μg/kg; Figure 1A) and a vagoinhibitory effect at high doses of 5-CT (50 and 100 μg/kg) (Figure 1A). On the contrary, i.v. bolus administration of neither α-methyl-5-HT (selective 5-HT_2_ receptor agonist) nor 1-PBG (the selective 5-HT_3_ receptor agonist) modified the decreases in electrically originated HR (Figure 1B).

**Figure 2:**
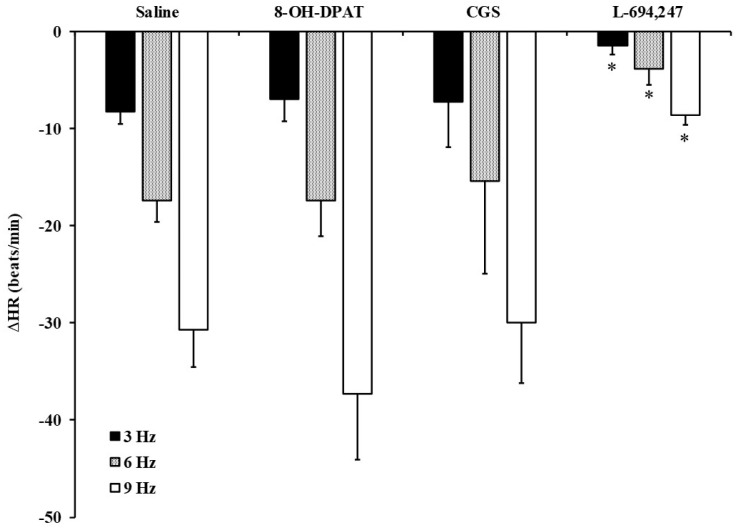
Effect of 5-HT1 receptor subtypes on the vagally-induced bradycardic responses in fluoxetine-treated diabetic rats. Effect of i.v. bolus injections of saline (1 mL/kg), 8-OH-DPAT, CGS-12066B (CGS) and L-694,247 (50 μg/kg each agonist; *n* = 5 for each treatment) on the decreases in heart rate (ΔHR) evoked by electrical vagal stimulation (3, 6 and 9 Hz) in fluoxetine-treated diabetic pithed rats. * *p* < 0.05 versus saline.

The inhibitory bradycardic effect induced by high doses of 5-HT (100 and 200 μg/kg; Table 2) and 5-CT (50 and 100 μg/kg; Figure 1) was reproduced by i.v. bolus of 50 μg/kg L-694,247 (a selective 5-HT_1D_ receptor agonist, Figure 2). However, CGS-12066B (5-HT_1B_ receptor agonist; 50 μg/kg), or 8-OH-DPAT (5-HT_1A_ receptor agonist; 50 μg/kg) did not affect the bradycardia elicited by electrical stimulation of the vagus nerve (Figure 2).

### 2.5. Influence of Saline, SB269970 or LY310762 on the 5-CT or L-694,247 Effect on the Electrical-Induced Bradycardic Responses in FT Diabetic Rats

**Figure 3:**
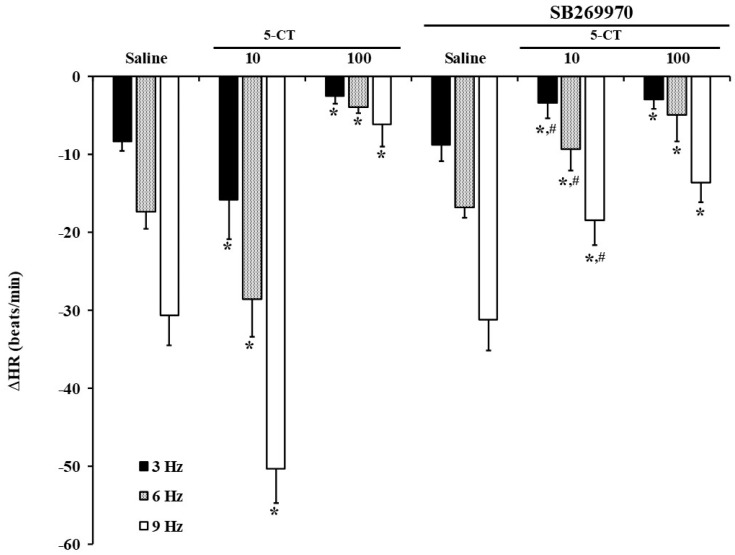
Effect of i.v. bolus of SB269970 in the saline- or 5-CT-influence on the electrically-induced bradycardic responses in fluoxetine-treated diabetic rats. Changes in decreases in heart rate (ΔHR) by electrical vagal stimulation after i.v. administration of bolus of saline (1 mL/kg) or 5-CT (10 and 100 μg/kg) in the presence or absence of SB269970 (0.5 mg/kg. i.v.) in fluoxetine-treated diabetic pithed rats. * *p* < 0.05 versus respective saline. # *p* < 0.05 versus 5-CT 10 μg/kg.

An exhaustive pharmacological insight was performed, analysing the role of several 5-HT antagonists in the 5-CT role on the electrical-induced bradycardia. The role of 5-HT_7_ receptor in the electrically-induced reductions of HR was studied, considering that 5-CT is a 5-HT_1/7_ receptor agonist and SB269970 is a selective 5-HT_7_ antagonist. Figure 3 shows that i.v. pretreatment with the selective 5-HT_7_ receptor antagonist, SB269970 (0.5 mg/kg) did not alter the inhibition of the bradycardia induced by 100 μg/kg of 5-CT. However, pretreatment with SB269970 made low doses of 5-CT (10 μg/kg) to manifest an inhibitory action. Thus, SB269970 completely blocked the potentiating action of low doses of 5-HT, demonstrating the role of 5-HT_7_ receptors in this action. 

**Figure 4:**
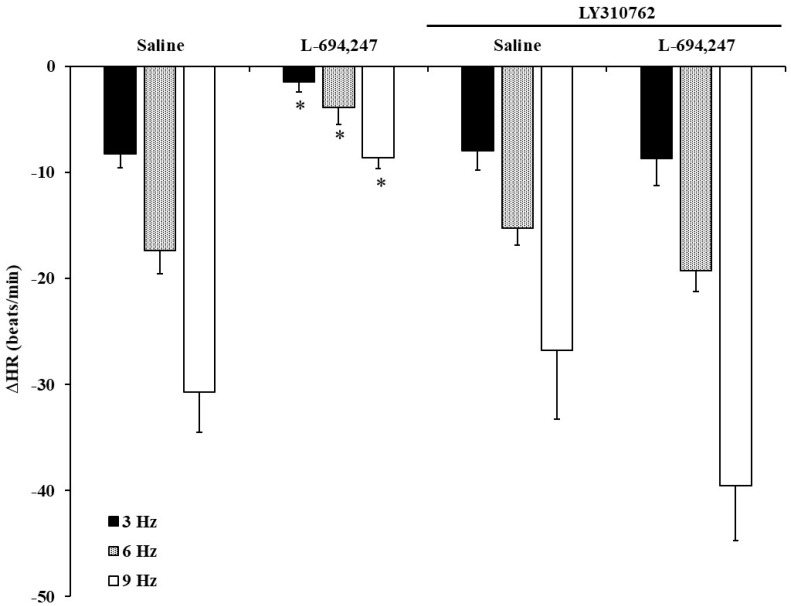
Effect of L-694,247 in the presence of 5-HT1D receptor antagonism on the electrically-induced decreases in heart rate in fluoxetine-treated diabetic rats. Effect of i.v. bolus injections of LY310762 (1 mg/kg) in the inhibitory effect of L-694,247 (50 μg/kg) on the decreases in heart rate (ΔHR) evoked by electrical vagal stimulation (3, 6 and 9 Hz) in fluoxetine-treated diabetic pithed rats. * *p* < 0.05 versus saline.

I.V. administration of LY310762, a selective 5-HT_1D_ receptor antagonist, prior to 50 μg/kg of L-694,247 completely blocked the inhibition of the vagally-induced bradycardia originated by this selective 5-HT_1D_ agonist in FT diabetic rats (Figure 4).

### 2.6. 5-CT Action on the Bradycardia Originated by Administration of Exogenous ACh (i.v.)

Furthermore, we tried to deepen in the nature of the response. To do so, we determined whether 5-CT would also alter the bradycardia provoked by i.v. bolus injections of exogenous ACh. Hence, as shown in Table 3, the bradycardia by administration of exogenous ACh (1–10 μg/kg, i.v.), which remained unchanged after saline (1 mL/kg, i.v.), was not significantly modified after i.v. administration of 10 and 100 μg/kg of 5-CT.

## 3. Discussion

Due to the increased prevalence of comorbid diabetes and depression, it becomes urgent to determine the mechanisms by which the most used treatment in depression (fluoxetine) may modify the evolution of microvascular diabetic complications. In this line, the role of fluoxetine treatment on the cardiac vagal input as well as on the serotonergic regulation of heart parasympathetic innervation during diabetes has not yet been elucidated. 

In our experiments, alloxan administration induced, from day 2 after injection, a syndrome resembling human T1DM [19,20,28] that was confirmed by measurement of blood glucose levels. Our animals were chronically treated with the antidepressant fluoxetine, which increases extracellular serotonin levels [29] at a dose and period (10 mg/kg/day; 14 days) that originate levels in plasma similar to patients treated with therapeutic doses [25,27,30,31,32]. Our results reveal that chronic fluoxetine treatment significantly reduces blood glucose levels compared to diseased (alloxan) animals without treatment, even though glycaemia does not return to normal levels, as we have previously described [27]. Similarly, body weight increase was significantly lower in FT compared to control diabetic rats [current results,27]. In this line, some studies revealed that fluoxetine can improve glucose tolerance and insulin resistance during diabetes [33] and, in non-diabetic patients, Ghaeli et al., [34] has reported that orally administered fluoxetine exhibits reduced levels of fasting blood glucose.

After anaesthesia and pithing, fluoxetine treatment did not modify basal MBP and HR compared to FT normoglycaemic [25,30] and non-treated diabetic rats (current data, [19,20,27]). While most of the agonists and antagonists used in this work did not modify either MBP or HR, it is remarkable that all doses of 5-HT and α-methyl-5-HT induced increases in MBP that immediately restored to basal values as occurred in FT normoglycaemic animals [25]. Due to its length, the vagus nerve is particularly vulnerable to long-term hyperglycaemia, and, therefore, vagal function is frequently affected early in the genesis and progression of CAN [5,35]; in fact, measurement of cardiac vagal tone has been suggested to be an identifier of CAN as it is significantly lower in patients suffering from T1DM [36]. Stimulation of the vagus nerve induces bradycardia in our experimental model. These responses are clearly due to release of ACh, since, as our previous works have shown, pretreatment with atropine completely abolished these bradycardic responses [19,21,25]. Antidepressant treatment (fluoxetine) in diabetic animals indirectly reveals a reduction of ACh release from the vagus nerve, since the bradycardic responses obtained by electrical stimulation are lower than in control diabetic rats [current data,19] and normoglycaemic rats [18], which may clearly influence the development of CAN.

The present work shows that, in FT diabetic rats, 5-HT has a pivotal effect in regulating parasympathetic cardiac tone, exerting both potentiating and inhibiting actions of bradycardic responses produced by electrical stimulation. Low doses of 5-HT (10 μg/kg) potentiate the bradycardia obtained by electrical stimulation whereas high doses (100 and 200 μg/kg) inhibit it. These results bring to light that chronic treatment with fluoxetine alters the serotonergic modulation of cardiac cholinergic drive in short-term diabetes [19]. However, fluoxetine influence on 5-HT modulatory action on electrically-induced bradycardia pointed out the dualism of serotonin effects already displayed in long-term diabetes [20]. As occurred in experimental models of long-term diabetes [20], the dual effect of serotonin is only reproduced by the selective 5-HT_1/7_ agonist, 5-CT, enhancing ACh release from the vagus nerve (measured as bradycardic responses) at low doses and diminishing it at high doses. These findings suggest that, in FT diabetic rats, the serotonergic effects on the electrically induced bradycardia are mediated through activation of the 5-HT_1/7_ receptors, but not through activation of the 5-HT_2_ or 5-HT_3_ receptors, as occurred in normoglycaemic rats [18].

To further test the receptors involved in the 5-CT effect, selective receptor agonists and antagonists were tested. As 5-CT has affinity not only for 5-HT_1_ receptor type, but also for 5-HT_7_ receptor type [21], the possible implication of 5-HT_7_ receptors was achieved by administering 5-CT at low and high doses in the presence of the selective 5-HT_7_ receptor antagonist, SB269970 [27,37]. SB269970 did not modify the inhibitory effect of low doses of 5-CT, but confirmed the involvement of 5-HT_7_ receptors in the high doses of 5-CT-mediated vagopotentiation, since in the presence of this antagonist, 5-CT manifested an inhibitory action. As already described, our current results confirm that the effector mechanism of 5-HT_7_ receptor (coupled to Gs proteins) is usually associated with an enhancement of neurotransmitter release [38,39], in this case, release of ACh from the vagus nerve. Remarkably, FT induces changes in the receptors involved in the enhancement of the ACh release produced by serotonin, since in diabetic rats, the receptors involved in the potentiating action were the 5-HT_1A_ receptors [19,20]. Moreover, in normoglycaemic animals chronically treated with either fluoxetine or sarpogrelate, 5-CT only exerted vagoinhibitory effects [21,25].

Neither 8-OH-DPAT nor CGS-12066B, selective 5-HT_1A_ and 5-HT_1B_ receptor agonists, respectively, were able to reproduce any serotonergic effect by 5-CT. However, the selective 5-HT_1D_ receptor agonist, L-694,247 reproduced the 5-CT vagoinhibitory effect, revealing that the 5-HT_1D_ receptor as involved in this inhibitory action; this is supported by the effector mechanism (through coupling to the Gi/o protein), reducing the release of neurotransmitters [39,40]. This point was assessed by the use of the selective 5-HT_1D_ receptor antagonist, LY310762, that completely abolished the L-694,247 inhibitory effect on the bradycardia. On the other hand, one might speculate on the locus of the 5-HT_1D/7_ receptors involved in this cardiac vagomodulation. As the model of pithed rat was utilised, central mechanisms can be excluded. It is noteworthy that 5-CT (at low and high doses) did not affect the bradycardia to exogenous ACh; hence, the most likely locus of the above 5-HT_1D/7_ receptors might be prejunctionally. The prejunctional nature of the 5-HT_1D/7_ receptors has been shown in other experimental models of chronic treatment in normoglycaemic rats [21,25]. However, it is noteworthy that in these cases, both 5-HT_1D_ and 5-HT_7_ exerted vagoinhibitory action.

Our study shows some constraints that should be taken into account: (1) there is no sex bias since, based in our prior experience, it was only performed in male animals; (2) central effects of fluoxetine are dodged as we utilised a pithed rat model; (3) despite the existence of a positive linear relationship between QTc interval prolongation and CAN severity in the diabetic population [41], indicating that prolonged QTc intervals are considered reliable predictors of heart disease and fatal ventricular arrhythmias [42,43,44], we did not perform electrocardiogram analysis in this study; and (4) vagal nerve activity is not directly measured, even though it is indirectly assessed by the bradycardia evoked by ACh release due to vagal stimulation. However, considering that nowadays, the growing numbers of comorbid diabetes mellitus and depression continues to be a challenging health problem, the clinical relevance of knowing how the most used worldwide antidepressant, fluoxetine, may affect the evolution of T1DM and its cardiovascular complications becomes a top-rated need in public health. In this sense, this study brings to light that the serotonergic system is a main actor as modulator of cholinergic input at peripheral level, specifically, at cardiac level, in fluoxetine-treated diabetic individuals. So, it is worth to indicate that this work in basic pharmacology put in relevance that, in fluoxetine-treated diabetic rats, activation of prejunctional 5-HT_7_ receptors increases cholinergic-evoked bradycardic responses, whereas they are inhibited by prejunctional 5-HT_1D_ receptors. Thus, the utilisation of selective 5-HT_7_ receptor agonists may augment parasympathetic drive in the heart and, therefore, may become a therapeutic target to tackle HR variability alterations that occur during CAN development in diabetic and depressive patients under fluoxetine treatment. 

Considering that (1) nowadays, a great number of diabetic patients suffer from anxiety or depression and are under fluoxetine treatment [45], (2) HR variability is diminished in diabetic patients due to an impairment of parasympathetic activity [6], which is a predictive tool for CAN, (3) chronic fluoxetine treatment in rats reduces bradycardic responses to endogenous ACh release (current data) and (4) the serotonergic system seems to be a main player in this match, not only at the central level to control depressive state, but also at the peripheral level, acting as a vagoinhibitor via prejunctional 5-HT_1D_ receptors, as well as a booster of bradycardia via prejunctional 5-HT_7_ receptors (present results), our results suggest that ACh release at cardiac level may be modulated using selective 5-HT receptor subtype agonists in these patients (FT diabetic individuals) to ameliorate the imbalance of sympathetic-parasympathetic systems that alter HR variability (as predictive of CAN). 

In conclusion, this study discloses that fourteen-day oral fluoxetine treatment in experimental diabetes originates a decrease in cholinergic activity at the cardiac level and changes the 5-HT regulation on cardiac parasympathetic drive in type 1 diabetic rats. Activation of prejunctional 5-HT_7_ receptors augments cholinergic-evoked bradycardic responses, whereas inhibition of vagally-induced bradycardia is mainly mediated by prejunctional 5-HT_1D_ receptors. Accordingly, this work contributes to the knowledge of complex mechanisms occurring in fluoxetine-treated diabetic (depressive) patients, opening the way to new therapeutic tools based on serotonergic system modulation of cardiac parasympathetic drive to tackle diabetic cardiac disturbances.

## 4. Materials and Methods

### 4.1. Drugs Used

The compounds used in the present study were obtained from the following sources: sodium pentobarbital (Dolethal^®^; Vetoquinol; Madrid, Spain); fluoxetine hydrochloride was from Normon (Madrid, Spain); heparin sodium from Rovi (Madrid, Spain); alloxan monohydrate, 5-HT, 1-phenylbiguanide (1-PBG), 7-trifluoromethyl-4-(4-methyl-1-piperazinyl)pyrrolol [1,2-a]-quinoxaline dimaleate (CGS-12066B), ACh chloride and atenolol were from Merck Life Sciences S.L.U. (Madrid, Spain); 5-carboxamidotryptamine maleate (5-CT), α-methyl-5-hydroxytryptamine maleate (α-methyl-5-HT), 8-hydroxy-2-dipropylaminotetralin hydrobromide (8-OH-DPAT), 2-[5-[3-(4-methylsulfonylamino)benzyl-1,2,4-oxadiazol-5-yl]-1H-indol-3-yl]ethanamine (L-694,247), (R)-3-[2-[2-(4-methylpiperidin-1-yl)ethyl]pyrrolidine-1-sulfonyl]phenol hydrochloride (SB269970) and 1-[2-[4-(4-fluorobenzoyl)-1-piperidinyl]ethyl]-1,3-dihydro-3,3-dimethyl-2H-indol-2-one hydrochloride (LY310762) were from Tocris Bioscience (Bristol, UK). All drugs were dissolved in physiological saline at the time of experimentation, which had no effect on basal MBP or HR. The doses of all drugs refer to their free base.

### 4.2. General Methods

Rats (*n* = 120) were made diabetic by a subcutaneous injection of alloxan (150 mg/kg) dissolved in physiological saline. Body weight was controlled, and non-fasting blood glucose levels were measured with a glucometer (Accu-chek^®^ Aviva; Roche Diagnostics; Barcelona, Spain) before and periodically after alloxan administration until day 28. Rats with blood glucose levels < 250 mg/dL (non-diabetic) were discarded. From days 14 to 28, animals were orally treated with fluoxetine (10 mg/kg/day, dissolved in drinking water; *n* = 115) [27]. Fluoxetine dose was adjusted every day and drinking bottles were protected from light. A set of five animals remained as the control diabetic group (without oral administration of fluoxetine).

**Figure 5:**
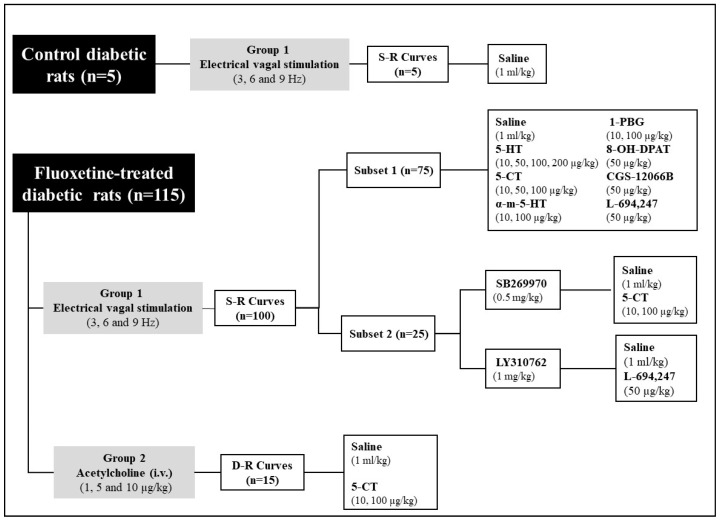
Experimental protocols showing the number of animals used. Experimental protocols showing the number of animals used in the control diabetic group and 2 main groups of fluoxetine-treated diabetic rats and in the different subsets used in the present study. Bradycardic responses were obtained by electrical vagal stimulation (groups 1 of control and fluoxetine-treated diabetic rats) or i.v. bolus of acetylcholine (group 2 of fluoxetine-treated diabetic rats). All drugs were administered as i.v. bolus.

After 28 days of diabetes induction, animals were anaesthetised (sodium pentobarbital, 60 mg/kg, i.p.) and pithed [18,19]. Jugular veins were catheterised for drug administration. MBP and HR were monitored through the left carotid artery, using the software Labchart Scope through a PRS 206 amplifier (Cibertec, Madrid, Spain) and a Power Lab System (AD Instruments, Oxford, United Kingdom). Vagus nerves were isolated and set for electrical vagal stimulation [18,19,20,25]. Animals were then pre-treated intravenously with heparin (1000 UI/kg;) and atenolol (1 mg/kg) [21,25]. Then, FT diabetic animals were split into 2 groups, (Figure 5) in order to analyse the role of several serotonergic agents on the bradycardic responses evoked by electrical vagal stimulation (group 1; *n* = 105) or by intravenous bolus injections of exogenous ACh in FT diabetic rats (group 2; *n* = 15).

The bradycardic S-R curves or D-R curves induced by electrical stimulation or exogenous ACh, respectively, were completed in about 15 min, with no significant changes in MBP. The electrical stimuli (3, 6 and 9 Hz; at 15 ± 3 V; 1 ms; for 15 s at 5-min intervals), as well as the i.v. bolus injections of exogenous ACh (1, 5 and 10 μg/kg), were given using a sequential schedule at 3- to 5-min intervals [18,19,20,25].

To prevent cumulative responses or tachyphylaxis, each animal was used to investigate only one dose of agonist or antagonist, so that only two (S-R or D-R) curves were obtained in each animal (i.e., control curve and treatment curve).

### 4.3. Experimental Design

After a 10-min period to stabilise haemodynamic conditions, MBP and HR were determined, and afterwards, animals were blindly separated to perform Protocol I and II.

#### 4.3.1. Protocol I: Vagus Nerve Electrical Stimulation

The control diabetic group of animals (*n* = 5; Figure 5), received i.v. bolus injection of saline (1 mL/kg, *n* = 5) and was used to confirm previous results by us [19].

The first group of FT diabetic rats served to study the function of serotonergic drugs on the electrically-evoked bradycardic responses [19,21,25], obtaining the first S-R curve (E0). The first subset of rats (*n* = 75; Figure 5), received i.v. bolus injections of: (i) saline (1 mL/kg, *n* = 5); (ii) 5-HT (10, 50, 100 and 200 μg/kg, *n* = 5 each); (iii) the 5-HT_1/7_ agonist, 5-CT (10, 50 and 100 μg/kg, *n* = 5 each dose); (iv) the 5-HT_2_ agonist, α-methyl-5-HT (10 and 100 μg/kg, *n* = 5 each); (v) the 5-HT_3_ agonist, 1-PBG (10 and 100 μg/kg, *n* = 5 each); (vi) the 5-HT_1A_ agonist, 8-OH-DPAT (50 μg/kg, *n* = 5); (vii) the 5-HT_1B_ agonist, CGS-12066B (50 μg/kg, *n* = 5); and (viii) the 5-HT_1D_ agonist, L-694,247 (50 μg/kg, *n* = 5). Five minutes after the administration, a new S-R curve (E1) was obtained. Then, a second subset was established to deepen on the serotonergic receptors that modulate the bradycardic responses. In this subset, animals received an i.v. bolus injection of SB269970 (5-HT_7_ receptor antagonist; 0.5 mg/kg) or LY310762 (5-HT_1D_ receptor antagonist; 1 mg/kg) respectively, performing the E0 antagonist curve after 10 min. Then, each of these pretreatments was thereafter subdivided into two subgroups that received an i.v. bolus injection of saline (1 mL/kg; *n* = 5) and 5-CT (10 and 100 μg/kg; *n* = 5 each) in animals pretreated with SB269970, and L-694,247 (50 μg/kg) or saline (1 mL/kg) in animals pretreated with LY310762 (*n* = 5 each). After 5 min, a new S-R curve was obtained (Figure 5).

#### 4.3.2. Protocol II: Intravenous Administration of Exogenous ACh

In the second group of FT diabetic animals, the platinum bipolar electrode was avoided; instead, i.v. bolus injections of exogenous ACh (1, 5 and 10 μg/kg) served to construct the D-R curves before (E’0) and 5 min after (E’1) i.v. administration (*n* = 5 each) of: (i) saline (1 mL/kg) and (ii) 5-CT (10 and 100 μg/kg) (Figure 5).

### 4.4. Statistical Analysis

Data are expressed as means ± SEM of at least five experiments (*n* = 5). Alterations on HR were expressed as decreases in beats/min from the corresponding baseline value. Statistical analysis between the experimental groups and their corresponding control group was performed with one-way ANOVA followed by the Student-Newman-Keuls’ post hoc test. Statistical significance was accepted at *p* < 0.05. Because the reductions in HR by i.v. vehicle (saline) were similar to those produced in the control group (receiving nothing), the statistical analysis was only performed versus saline.

## Figures and Tables

**Table 1:** Effect of 5-HT receptor agonists on haemodynamic variables in fluoxetine-treated diabetic rats.

5-HT Receptor Agonists (μg/kg)	ΔMBP (mm Hg)	ΔHR (beats/min)
5-HT (10)	39.2 ± 2.6 *	7.3 ± 2.4
5-HT (100)	76.1 ± 4.3 *	6.7 ± 1.2
5-HT (200)	89.5 ± 1.2 *	2.1 ± 0.6
5-CT (10)	−2.1 ± 0.6	3.4 ± 1.7
5-CT (100)	−6.5 ± 1.7	4.6 ± 1.5
α- m-5-HT (10)	42.2 ± 7.1 *	3.7 ± 0.8
α- m-5-HT (100)	86.4 ± 5.6 *	5.4 ± 2.1
1-PBG (10)	3.2 ± 0.9	3.2 ± 2.4
1-PBG (100)	6.1 ± 1.7	9.3 ± 4.9

Variations in baseline values of mean blood pressure (ΔMBP) and heart rate (ΔHR) after i.v. bolus administration of 5-HT (10, 100 and 200 μg/kg), 5-carboxamidotryptamine (5-CT; 10 and 100 μg/kg), α-methyl-5-HT (α-m-5-HT; 10 and 100 μg/kg) and 1-phenylbiguanide (1-PBG; 10 and 100 μg/kg) in fluoxetine-treated diabetic rats. All values are expressed as mean ± SEM. * *p* < 0.05 versus baseline.

**Table 2:** Effect of 5-HT on the electrically-induced bradycardia in fluoxetine-treated diabetic rats.

5-HT (μg/kg)	Vagal Stimulation Frequencies		
	3 Hz	6 Hz	9 Hz
Saline	−8.3 ± 1.3	−17.4 ± 2.2	−30.7 ± 3.8
10	−16.0 ± 2.0 *	−30.6 ± 6.2 *	−60.2 ± 7.5 *
50	−10.8 ± 8.5	−23.6 ± 8.2	−47.4 ± 8.8 *
100	−5.1 ± 1.2 *	−9.3 ± 1.7 *	−20.6 ± 3.2 *
200	−3.3 ± 1.1 *	−9.4 ± 3.1 *	−13.8 ± 1.0 *
	**ΔHR (beats/min)**		

Effect of i.v. bolus injections of saline (1 mL/kg) or 5-HT (10, 50, 100 and 200 μg/kg; *n* = 5 for each treatment) on the decreases in heart rate (ΔHR) evoked by electrical vagal stimulation (3, 6 and 9 Hz) in fluoxetine-treated diabetic pithed rats. * *p* < 0.05 versus saline.

**Table 3:** Effect of 5-CT on the acetylcholine-induced bradycardia in fluoxetine-treated diabetic rats.

ACh (μg/kg)	Saline (ml/kg)	5-CT (μg/kg)	
	1	10	100
**1**	−38.2 ± 3.3	−41.6 ± 5.7	−42.2 ± 2.1
**5**	−47.9 ± 6.1	−50.1 ± 5.4	−56.2 ± 4.1
**10**	−72.2 ± 4.7	−69.8 ± 6.3	−68.1 ± 3.9
	**ΔHR (beats/min)**		

Effect of i.v. bolus injections of saline (control group; 1 mL/kg) and 5-CT (10 and 100 μg/kg each) on the bradycardic responses (ΔHR) elicited by increasing i.v. doses of exogenous acetylcholine (ACh; 1, 5 and 10 μg/kg) in fluoxetine-treated diabetic pithed rats. No statistical differences.

## Data Availability

The main data are included in this manuscript. All data are available from the corresponding author on reasonable request.
